# Effects of a Dynamic Chair on Chair Seat Motion and Trunk Muscle Activity during Office Tasks and Task Transitions

**DOI:** 10.3390/ijerph15122723

**Published:** 2018-12-03

**Authors:** Corina Nüesch, Jan-Niklas Kreppke, Annegret Mündermann, Lars Donath

**Affiliations:** 1 Department of Orthopaedics and Traumatology, University Hospital Basel, 4031 Basel, Switzerland; corina.nueesch@usb.ch; 2 Department of Biomedical Engineering, University of Basel, 4123 Allschwil, Switzerland; annegret.muendermann@unibas.ch; 3 Department of Sport, Exercise and Health, University of Basel, 4052 Basel, Switzerland; jan-niklas.kreppke@stud.unibas.ch; 4 Department of Intervention Research in Exercise Training, German Sport University Cologne, 50933 Köln, Germany

**Keywords:** dynamic sitting, office chair, muscle activation, inactivity, sedentarism

## Abstract

Employing dynamic office chairs might increase the physical (micro-) activity during prolonged office sitting. We investigated whether a dynamic BioSwing^®^ chair increases chair sway and alters trunk muscle activation. Twenty-six healthy young adults performed four office tasks (reading, calling, typing, hand writing) and transitions between these tasks while sitting on a dynamic and on a static office chair. For all task-transitions, chair sway was higher in the dynamic condition (*p* < 0.05). Muscle activation changes were small with lower mean activity of the left obliquus internus during hand writing (*p* = 0.07), lower mean activity of the right erector spinae during the task-transition calling to hand writing (*p* = 0.036), and higher mean activity of the left erector spinae during the task-transition reading to calling (*p* = 0.07) on the dynamic chair. These results indicate that an increased BioSwing^®^ chair sway only selectively alters trunk muscle activation. Adjustments of chair properties (i.e., swinging elements, foot positioning) are recommended.

## 1. Introduction

Physical inactivity is considered an independent risk factor for many non-communicable diseases and has been entitled as biggest public health problem of the 21st century [1]. Projections have indicated that the metabolic equivalent (MET) of daily physical work will drop to 190 MET hours per week in 2030 because of technological development and automation [2]. In comparison, 24 h of sleeping every day corresponds to approximately 150 MET hours per week [2]. Today, most persons in developed countries spend more than half of their waking hours in seated postures which negatively affects mortality and increases the risk of, for instance, cardiovascular disease, diabetes, back pain, stroke, and obesity [3]. Hence, it is important to not only increase leisure time physical activity (PA) but also possibly increase muscle activations during sitting.

Besides leisure time PA opportunities, work places involving mainly sitting activities (e.g., office, libraries, lecture halls) represent a challenging but also promising setting for encouraging PA. In recent years, researchers have focused on active breaks, cycling desks and standing desks for promoting occupational PA [4,5,6]. However, despite of promising evidence such approaches have not been widely implemented. Another strategy for attaining greater PA at work, is altering the chair design aiming to increase “micro-activity” of the trunk while sitting [7]. Previous studies showed increased muscle activation in either thoracic or lumbar erector spinae during sitting on a stability ball [8] while performing office tasks but not for sitting on dynamic chairs [7,9]. However, meanwhile, more sophisticated dynamic chairs with moving seats have been developed. For instance, the BioSwing^®^ chair (Haider Bioswing, Pullenreuth, Germany) enables two-dimensional swinging of the seating area. These reflective micro-motions of the trunk induced by the body are believed to beneficially affect trunk motion and muscle activity, yet to date evidence of this mechanism is lacking. Moreover, typical office activities do not only comprise individual tasks but also transitions between tasks. It has been shown that the muscle activation level during sitting is task-dependent [7]. Task transitions (i.e., from typing on the computer to calling on the phone) require more movement and can be very frequent during office works with up to 30 or 140 transitions per hour in some workers depending on the measurement method [10]. Thus, it is possible that differences in muscle activation patterns are more pronounced for tasks transitions than individual tasks. The main purpose of this study was to investigate whether dynamic sitting on a BioSwing^®^ chair increases sway of the chair seat. Secondly, we tested the hypothesis that a greater sway of the chair induces relevant changes in trunk muscle activation during typical office tasks (reading, hand writing, calling, typing on the keyboard) and their task-transitions. Such “micro-activity” of the trunk may be particularly important when prolonged sitting cannot be avoided or when habits cannot be changed.

## 2. Materials and Methods

### 2.1. Participants

Twenty-six healthy young adults (11 males, 15 females; age 23.4 ± 1.9 years; body mass 65.9 ± 10.5 kg; height 1.73 ± 0.08 m; body mass index 21.9 ± 2.6 kg/m^2^) participated in this study. Written informed consent was given prior to the start of the study. Participants were blinded the chair conditions and no information on the chairs were provided. Inclusion criteria were age between 18 and 55 years of age, working most of the time in sitting position (i.e., as office workers or students), and proficiency in German language for correct and reliable introductions of the tasks. Exclusion criteria were pain, restrictions in sitting position and pregnancy. All participants were right handed and used their right hand for both writing and calling.

### 2.2. Experimental Setup and Study Design

**Figure 1:**
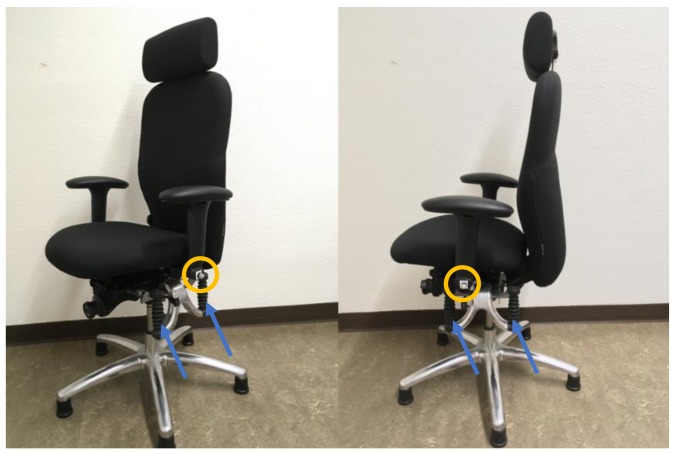
The dynamic swing system is located beneath the seat of the office chair (Haider Bioswing, Pullenreuth, Germany). The blue arrows indicate the location of two of four swinging elements. The yellow circles illustrate the location of the reflective markers at the base of the arm rest.

The study design and applied procedures complied with ethical standards and were approved by the regional ethics committee (approval number: EKNZ 2016-01903). We implemented a single randomized crossover-design for sitting on two different Haider Bioswing office chairs (Figure 1). Both chairs looked identical, but in one chair, the swinging system under the seat was unlocked (dynamic chair), while in the other chair the swinging system was locked and stable (static chair). The swinging system facilitates for motion in the seat plane by employing swinging and damping elements. Both chairs were equipped with feet that did not allow motion between the chair and the floor. The participants were not aware of the differences between the chairs.

**Figure 2:**
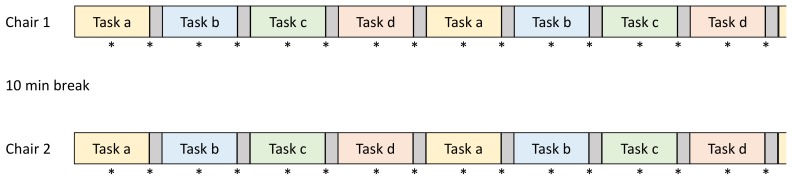
Overview of the measurement procedures. Each start of a kinematic and EMG (electromyography) measurement is indicated with an asterisk (*). The grey areas between two tasks indicate the 30 s period of each task transition. Tasks a to d correspond to reading, calling, typing and hand writing with the order of the tasks being randomized.

For each chair condition, participants were asked to complete four different tasks in randomized order: reading on a computer; typing on a keyboard; writing by hand; and a phone call using a mobile phone. Each task was performed for 3.5 min followed by a 30-s task-transition period where the participants were instructed for the new task. The same sequence of the four tasks was performed twice on each chair resulting in a total sitting time of 32 min per chair. The order of the chair conditions was randomly assigned. There was a 10-min break between testing sessions on the two chairs. Trunk muscle activity and movement of the chair were measured during the middle 30 s of the task and during the 30-s task-transition using surface electromyography and reflective markers (Figure 2).

All tests were performed under standardized conditions in an indoor laboratory. Chair height was adjusted for each participant to ensure full contact of the feet with the floor and a knee angle of 90° and was identical between chairs.

### 2.3. Tasks and Task Transitions

For every participant, identical instructions for the tasks and task transitions were used. The task “typing on a keyboard” consisted of writing a text about the participant’s current workplace (required tasks, preferences, dislikes and future possibilities). During the task “reading on a computer”, participants were always asked to first open a text file and then read a text about office work. The task “writing by hand” consisted of writing a text about short-term and long-term goals and their implementation. For the task “calling”, participants were instructed to enter the number on their mobile phone and to hold the phone to their ear while talking about the participants’ field of work and work tasks.

### 2.4. Kinematics

Chair motion and upper body position were measured using an 8-camera motion analysis system (Vicon, Oxford, UK). Reflective markers were placed at the base of the left and right armrest representing the chair center (Figure 2) and on the jugular notch representing the trunk. The length of the path of the midpoint between the two markers on the chair (chair center) and of the marker on the jugular notch (trunk) was calculated and used for further analysis of chair and trunk motion.

### 2.5. Electromyography

After shaving and cleaning the skin with alcohol, six wireless surface electromyography (EMG) electrodes (Myon AG, Schwarzenberg, Switzerland) were placed bilaterally on the erector spinae, obliquus internus abdominis and rectus abdominis muscles according to the guidelines of the European Surface Electromyographic Society (SENIAM) [11,12].

To remove electrocardiogram (ECG) contamination in the EMG signal of the trunk muscles, an independent component analysis based ECG filter was used [13]. All EMG signals were bandpass filtered (4th order Butterworth filter, 10–450 Hz) to remove movement artifacts and high frequency noise. EMG envelopes were calculated with a moving average filter and a window size of 41.7 ms (100 data points). For each participant and muscle, the mean intensity during each task and task transition was calculated. For the two chair conditions, the average of the two repetitions of each task and task transition was used for further analysis.

### 2.6. Statistics

Differences in the length of path of the chair center and mean muscle activation levels between the two chair conditions were analyzed using paired *t*-tests. Effect sizes were estimated using Cohen’s d [14]. Calculations were performed in Matlab (2017a, MathWorks, Natick, MA, USA).

The change scores between both chairs were calculated as the 90% confidence intervals according to the magnitude-based inference approach [15]. A practically worthwhile change was assumed when the difference score was at least 0.2 of the between-subject standard deviation [16]. The probability for an effect being practically worthwhile was calculated according to the magnitude-based inference approach using the following scale: 25–75%, possibly; >75%, likely; >95%, very likely; >99.5%, most likely [15]. The default probabilities for declaring an effect practically beneficial were <0.5% (most unlikely) for harm and >25% (possibly) for benefit [16]. Calculations were conducted using a Microsoft^®^ Excel spreadsheet (Redmond, WA, USA) [17].

## 3. Results

### 3.1. Chair and Trunk Motion

**Figure 3:**
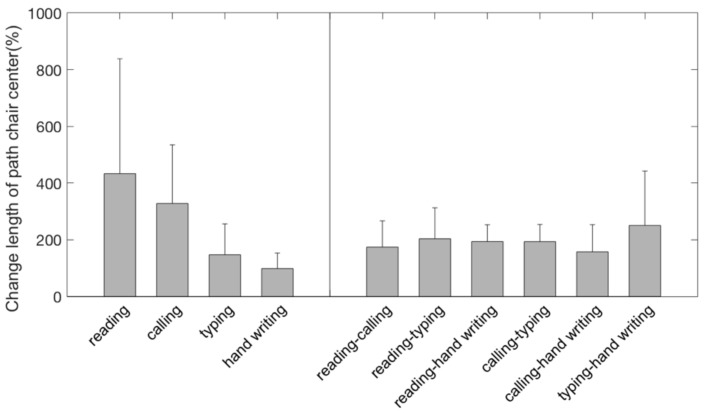
Relative increase in path length of the chair center for sitting on the dynamic chair compared to the static chair for the different tasks and task transitions.

On average, the length of path of the chair center for the dynamic chair was between 100 and 400% longer for the individual tasks and around 200% greater for the task transitions compared to the static chair with a high between subject variability (Figure 3). For the separate tasks, this difference was statistically significant during calling but not during the other three tasks (Table 1). For all task transitions, this difference was statistically significant with higher effect sizes (Table 1).

**Figure 4:**
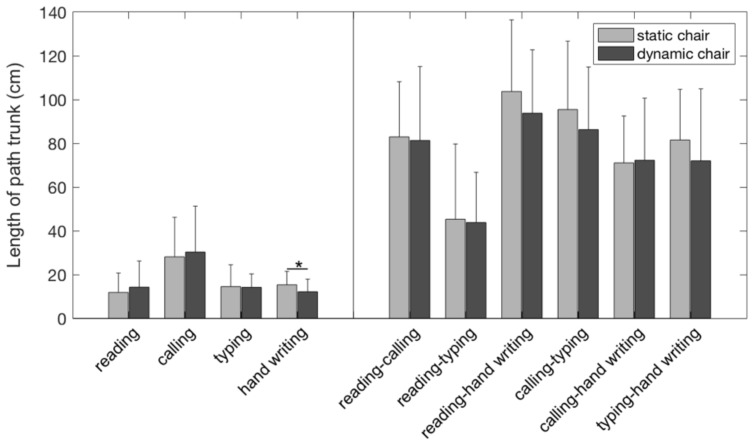
Length of path of the trunk motion during the different tasks and task transitions while sitting on the dynamic (dark grey) and static chair (light grey). The asterisk indicates (*) significant differences between the chair conditions (*p* < 0.05).

The length of path of the trunk was on average shorter on the dynamic compared to the static chair during hand writing (*p* = 0.041, effect size = 0.349). For the other tasks and all task transitions, there were no significant differences in the length of path between the chairs (Figure 4).

### 3.2. Muscle Activation

#### 3.2.1. Tasks

Mean muscle intensities while performing the different tasks did not differ between the dynamic and static chairs. Yet for hand writing, the left obliquus internus muscle showed a possible effect with lower mean intensity on the dynamic chair than on the static chair with only a small effect (*p* = 0.066; effect size = −0.188; Table 2).

#### 3.2.2. Task transitions

For the transition from reading to calling, the mean intensity of the left erector spinae was likely higher for the dynamic chair than the static chair (*p* = 0.067; effect size = 0.332). For the transition from calling to hand writing, the mean intensity of the right erector spinae was likely lower for the dynamic than the static chair (*p* = 0.036; effect size = −0.192). Moreover, there was a possibly lower mean intensity of the right erector spinae during the transition from reading to hand writing and typing to hand writing, of the left obliquus internus during the transition from calling to hand writing, of the left rectus abdominis during the transition from reading to typing, and of the right rectus abdominis during the transition from calling to hand writing for the dynamic chair compared to the static chair. All observed effect sizes were small (Table 3).

## 4. Discussion

The aim of this study was to investigate whether a BioSwing^®^ office chair induces changes in chair motion and trunk muscle activity compared to a static non-swinging chair in a single blinded randomized controlled crossover trial. Our results indicate that (a) the path length of the chair center was significantly longer for the dynamic chair during calling and all task transitions and (b) muscle activation during all tasks did not differ between the chairs but showed small to notable changes during the task transitions reading to calling, calling to writing and typing to writing. Specifically, for the transition between reading and calling bilateral erector spinae muscle activation was higher for the dynamic chair than for the static chair but effect sizes were small and MBI revealed only a trivial effect. For the transitions between calling and writing and between typing and writing the right erector spinae muscle activation was lower for the dynamic chair than for the static chair with very small effect sizes and trivial MBI.

### 4.1. Chair Center

The longer path length of the chair center for the dynamic than for the static chair confirms that the springs facilitate motion of the seat. This is comparable to previous results with a different dynamic office chair that showed greater chair movements (measured on the top of the backrest) when performing office tasks such as typing on a keyboard and placing files in folders while sitting on a dynamic compared to a conventional chair [18]. During all measurements, participants had their feet on the floor, which typically has a stabilizing effect on the body especially for more static tasks such as writing by hand, typing and reading on the laptop screen where the arms are also placed on the desk. Thus, limited movement of the hips and buttocks might result in negligible changes in chair movement and trunk muscle activation. However, motion analyses of the hip and buttocks were not explicitly conducted. During the condition calling, participants moved more freely on the chair, often sitting back in the chair and not placing their arms on the desk thereby increasing the degrees of freedom of possible motion which has been shown to increase postural sway [19]. Moreover, this might allow more micro-motions of the seat of the dynamic chair than the static chair resulting in a longer path length of the chair center. Similarly, when performing task transitions, participants often had to remove their arms from the desk and move in the chair enabling the swinging of the seating area as seen in the longer path length of the chair center for the dynamic chair than for the static chair.

### 4.2. Muscle Activation

Previous studies on trunk muscle activation during sitting on different chairs reported different changes in muscle activation levels depending on the design of the tested chairs and measured muscles. No differences in lumbar and thoracic erector spinae activation were reported for reading a book, word-processing and computer-aided design between sitting on a chair with fixed seat and backrest, a chair which allowed independent sagittal plane rotation of the seat and back rest and a chair which allowed a rotation in a fixed ratio between the seat and back rest [7]. Similarly, Ellegast et al. did not observe an effect of sitting on five different office chairs on erector spinae muscle activation during different tasks such as typing, calling, and reading and correcting text data [9]. However, the office chairs investigated in these studies differed from the office chairs tested in our study. Nonetheless, it is possible that the variability in performing different office tasks between trials and persons is higher than the difference in the construction of the office chairs. On the contrary, sitting on a stability ball resulted on average in a higher activation of the left thoracic erector spinae muscle during reading, computer-aided design and typing than sitting on a standard office chair without armrests [8]. However, participants in that study also reported an increased level of discomfort, which might be related to altered posture or muscle activations. An ergonomic backless chair resulted in a significantly lower activation of the iliocostalis pars thoracis muscle for a typing task compared to a standard backless office chair [12]. In a systematic review, it was concluded that the changes in muscle activation levels during dynamic sitting may be attributable to the absence of a backrest in some dynamic chairs [20]. However, because the BioSwing^®^ chair used in our study has a backrest, the observed changes in muscle activation are likely attributable to the dynamic swinging system.

Generally, muscle activation levels during seated office tasks were small (below 10% of maximal voluntary contraction) in all studies [7,9]. Although we did not normalize muscle activation levels to maximal voluntary contractions but assessed change scores, visual inspections of the EMG signals showed that the signals remained close to baseline. None of the previous studies [20] reported effects of the different chairs on the activation of ventral trunk muscles (rectus abdominis, external oblique, internal oblique), which was confirmed for individual tasks for the dynamic BioSwing^®^ chair in our study. In contrast to these previous studies [20], our study showed differences in trunk muscle activations during task transitions. Task transitions comprise the combination of two tasks and include more movement and variability because the participants also rearranged the work space (i.e., moving laptop closer or away, picking up the phone). This may explain the small observed changes in the trunk muscles and mainly the erector spinae muscle. We are not aware of previous studies investigating task transitions, but effects of different office tasks on muscle activation levels during sitting have been reported [8,9].

Contrary to our initial hypothesis, we observed mainly possible or likely lower trunk muscle activity during task transitions while sitting on the dynamic compared to the static office chair. This result is in contrast to a previous study that showed an increase in the thoracic erector spinae muscle activation for sitting on an unstable ball compared to a conventional chair [8] but agrees with results from another study reporting lower activation of the iliocostalis pars thoracis muscle for a dynamic chair than a static chair without backrests [12]. It is possible that the swinging system of the dynamic chair facilitates lumbar trunk movements, thus reducing muscle activation levels and delaying a possible onset of fatigue.

### 4.3. Strengths and Limitations

The tested dynamic and static chairs in this study were identical except of the swinging mechanism being enabled for the dynamic and disabled for the static chair. Moreover, participants were blinded to the differences between chairs before the experiment to reduce potential conscious adaptions in sitting posture and movements during the measurements. To reflect common working situations, we not only investigated muscle activations during the performance of single tasks but also during task transitions. The randomization of the order of the tasks resulted in 12 possible task transitions with not every participant performing the same task transitions. To increase the number of observations per task transition, both directions of the task transition (i.e., calling to typing and typing to calling) were pooled for the analysis. This might have increased the variability in muscle activation patterns but should have little effects on mean muscle intensity over the entire 30-s measurement. Nevertheless, it is possible that the data pooling reduced the observed differences and resulted in smaller differences between chair conditions for task transitions. In this study, we tested the experimental chairs in 26 subjects. Similar results would have to be confirmed in a larger population. Moreover, the study population consisted of healthy young adults, and hence it is not clear if older adults or persons with back problems would experience similar differences. Nonetheless, the data presented in this study represents reference data for future studies on the effect of office chair design on trunk muscle activity chair motion.

## 5. Conclusions

The increase in the path length of the chair center of the dynamic chair indicates changes in the chair motion of the dynamic chair compared to those of the static chair. However, the observed altered chair motion was associated with only small differences in trunk muscle activation levels for sitting on the dynamic chair compared to the static chair. In addition to previous studies, not only office tasks but also tasks transitions were investigated and it was seen that there are more changes in trunk muscle activation levels during these task transitions than for the individual tasks. Hence, investigating task transitions might be more sensitive in assessing effects of dynamic chairs on muscle activation.

Greater changes in muscle activation levels may be achieved when removing the stabilizing effect of placing the feet on the floor by adding a foot rest facilitating swinging of the whole body. The effect of the dynamic chair on chair motion and muscle activity levels may depend on the stiffness of the swinging elements, and it may be necessary to adjust the stiffness of the swinging elements to the user’s body weight. Nonetheless, from an epidemiological perspective even small changes in muscle activity during office tasks and task transition may be meaningful in the long run considering that many professionals perform mainly seated tasks during their work life over decades.

## Figures and Tables

**Table 1:** Mean (1 standard deviation) length of path of the chair center while sitting on the static or dynamic chair performing the different tasks and task transitions.

Length of Path of Chair Center (cm)	Dynamic Chair Mean (SD)	Static Chair Mean (SD)	*p*-Value	Effect Size	MBI
Tasks					
Reading	1.2 (1.7)	0.6 (0.8)	0.111	0.444	likely
Calling	6.0 (5.6)	3.8 (4.4)	0.003	0.446	most likely
Typing	1.2 (1.4)	0.7 (0.7)	0.111	0.401	likely
Hand writing	0.9 (1.0)	0.6 (0.6)	0.096	0.349	likely
Task transitions					
Reading-calling	6.9 (5.4)	3.6 (2.6)	0.025	0.767	most likely
Reading-typing	4.5 (5.1)	1.7 (1.4)	0.016	0.745	most likely
Reading-hand writing	9.4 (5.8)	4.1 (3.1)	<0.001	1.150	most likely
Calling-typing	8.6 (6.0)	4.3 (3.6)	0.004	0.875	most likely
Calling-hand writing	4.5 (4.2)	2.0 (1.4)	0.012	0.800	likely
Typing-hand writing	7.2 (5.6)	3.8 (2.8)	0.047	0.766	most likely

SD—standard deviation; MBI—magnitude based inferences: the probability of a beneficial effect towards the dynamic chair.

**Table 2:** Mean (1 standard deviation) mean muscle intensity while sitting on the static or dynamic chair performing the different tasks.

Mean Intensity of Muscle (mV)	Dynamic Chair Mean (SD)	Static Chair Mean (SD)	*p*-Value	Effect Size	MBI
Reading					
Rectus abdominis right	14.4 (4.4)	14.2 (5.0)	0.824	0.044	trivial
Obliquus internus right	14.1 (4.6)	13.9 (4.8)	0.598	0.052	trivial
Erector spinae right	14.2 (5.0)	14.1 (5.6)	0.951	0.005	trivial
Rectus abdominis left	13.8 (4.1)	13.7 (4.5)	0.919	0.010	trivial
Obliquus internus left	14.1 (4.4)	14.4 (4.6)	0.613	−0.058	trivial
Erector spinae left	13.9 (5.0)	14.2 (5.7)	0.501	−0.054	trivial
Calling					
Rectus abdominis right	14.6 (5.0)	14.1 (4.8)	0.677	0.101	trivial
Obliquus internus right	14.6 (4.6)	14.7 (5.0)	0.858	−0.014	trivial
Erector spinae right	13.8 (4.7)	14.3 (5.3)	0.292	−0.105	trivial
Rectus abdominis left	13.5 (4.7)	14.0 (5.1)	0.243	−0.101	trivial
Obliquus internus left	15.5 (4.6)	15.2 (4.6)	0.483	0.073	trivial
Erector spinae left	14.3 (6.2)	15.1 (7.2)	0.380	−0.124	trivial
Typing					
Rectus abdominis right	14.1 (4.0)	14.0 (4.9)	0.863	0.021	trivial
Obliquus internus right	14.3 (4.5)	14.5 (4.8)	0.648	−0.036	trivial
Erector spinae right	15.3 (6.3)	15.7 (6.2)	0.699	−0.058	trivial
Rectus abdominis left	14.0 (4.6)	13.8 (4.8)	0.716	0.042	trivial
Obliquus internus left	14.4 (4.4)	14.6 (4.4)	0.655	−0.041	trivial
Erector spinae left	15.3 (7.0)	15.0 (6.7)	0.689	0.042	trivial
Hand writing					
Rectus abdominis right	13.9 (4.1)	14.2 (4.9)	0.421	−0.078	trivial
Obliquus internus right	14.3 (4.3)	14.8 (4.7)	0.131	−0.111	trivial
Erector spinae right	14.3 (5.0)	14.8 (5.5)	0.193	−0.105	trivial
Rectus abdominis left	15.3 (8.7)	14.6 (4.8)	0.567	0.097	trivial
Obliquus internus left	14.7 (4.2)	15.4 (4.1)	0.066	−0.188	possibly
Erector spinae left	15.7 (7.4)	16.0 (7.5)	0.603	−0.042	trivial

SD—standard deviation; MBI—magnitude based inferences: the probability of a beneficial effect towards the dynamic chair.

**Table 3:** Mean (SD) mean muscle intensity while sitting on the two chairs during the different task transitions.

Mean Intensity of Muscle (mV)	Dynamic Chair Mean (SD)	Static Chair Mean (SD)	*p*-Value	Effect Size	MBI
Reading to calling					
Rectus abdominis right	13.7 (4.5)	13.0 (5.3)	0.530	0.136	trivial
Obliquus internus right	13.7 (4.9)	13.6 (5.4)	0.844	0.020	trivial
Erector spinae right	16.3 (5.5)	15.5 (5.9)	0.336	0.132	trivial
Rectus abdominis left	13.5 (5.0)	13.8 (6.0)	0.609	−0.055	trivial
Obliquus internus left	14.7 (4.6)	13.8 (5.3)	0.224	0.179	trivial
Erector spinae left	17.0 (6.5)	15.0 (5.8)	0.067	0.332	likely
Reading to typing					
Rectus abdominis right	13.5 (4.7)	14.0 (5.0)	0.374	−0.095	trivial
Obliquus internus right	14.1 (4.8)	14.5 (5.1)	0.383	−0.078	trivial
Erector spinae right	14.6 (5.4)	15.3 (5.9)	0.251	−0.117	unclear
Rectus abdominis left	13.0 (4.5)	14.0 (4.9)	0.130	−0.199	possibly
Obliquus internus left	14.5 (4.1)	14.7 (5.0)	0.658	−0.054	trivial
Erector spinae left	14.4 (6.2)	15.1 (5.5)	0.317	−0.111	trivial
Reading to hand writing					
Rectus abdominis right	15.4 (1.9)	15.9 (4.5)	0.591	−0.143	trivial
Obliquus internus right	15.7 (3.0)	16.1 (3.7)	0.417	−0.120	trivial
Erector spinae right	17.9 (5.0)	18.9 (4.4)	0.139	−0.220	possibly
Rectus abdominis left	16.0 (6.1)	15.5 (4.1)	0.842	0.090	trivial
Obliquus internus left	16.3 (2.7)	16.6 (3.1)	0.468	−0.098	trivial
Erector spinae left	17.7 (5.9)	18.5 (4.9)	0.361	−0.162	trivial
Calling to typing					
Rectus abdominis right	16.3 (3.6)	15.4 (4.0)	0.554	0.247	trivial
Obliquus internus right	15.5 (3.1)	15.9 (3.8)	0.461	−0.106	trivial
Erector spinae right	18.4 (6.3)	18.0 (4.6)	0.571	0.081	trivial
Rectus abdominis left	15.4 (5.6)	14.8 (4.1)	0.677	0.132	trivial
Obliquus internus left	15.9 (2.5)	16.1 (3.4)	0.810	−0.041	trivial
Erector spinae left	19.4 (7.8)	18.9 (5.8)	0.620	0.073	trivial
Calling to hand writing					
Rectus abdominis right	13.6 (4.3)	15.2 (6.6)	0.121	−0.281	possibly
Obliquus internus right	14.5 (4.4)	14.8 (4.8)	0.217	−0.078	unclear
Erector spinae right	14.4 (5.6)	15.5 (5.8)	0.036	−0.192	likely
Rectus abdominis left	13.6 (4.2)	14.1 (4.4)	0.227	−0.120	unclear
Obliquus internus left	15.0 (4.0)	15.6 (4.6)	0.150	−0.124	possibly
Erector spinae left	16.1 (5.7)	15.8 (6.3)	0.642	0.053	trivial
Typing to hand writing					
Rectus abdominis right	13.4 (4.6)	13.4 (5.5)	0.978	−0.004	trivial
Obliquus internus right	13.7 (5.0)	14.3 (5.5)	0.247	−0.118	unclear
Erector spinae right	16.3 (5.8)	17.6 (6.3)	0.116	−0.207	possibly
Rectus abdominis left	13.1 (5.2)	14.2 (5.8)	0.213	−0.192	unclear
Obliquus internus left	14.3 (4.7)	14.7 (4.8)	0.431	−0.088	trivial
Erector spinae left	16.2 (6.1)	16.6 (6.1)	0.550	−0.073	trivial

SD—standard deviation; MBI—magnitude based inferences: the probability of a beneficial effect towards the dynamic chair.
